# Clinical efficacy of neuromodulation in isolated dystonia: A systematic review of motor function improvement

**DOI:** 10.1007/s10072-026-09011-6

**Published:** 2026-04-07

**Authors:** Diamante Carta, Viviana Lo Buono, Laura Culicetto, Salvatore Bertino, Carmen Terranova, Caterina Formica, Angelo Quartarone, Silvia Marino

**Affiliations:** 1 IRCCS Centro Neurolesi “Bonino Pulejo”, S.S. 113 Via Palermo C/da Casazza, Messina, 98123 Italy; 2 Department of Clinical and Experimental Medicine, University of Messina, Messina, Italy

**Keywords:** Deep brain stimulation, Dystonia, GPi, Motor improvement, MRgFUS, Non-invasive neuromodulation, Quetsi, STN, tDCS, Transcranial magnetic stimulation

## Abstract

**Background:**

Isolated dystonia is a disabling movement disorder for which neuromodulation represents a key therapeutic option in medically refractory cases. Deep brain stimulation (DBS) of the globus pallidus internus (GPi) is the current standard target, while subthalamic nucleus (STN) stimulation and non-invasive techniques such as repetitive transcranial magnetic stimulation (rTMS) and transcranial direct current stimulation (tDCS) have been investigated as alternative or adjunctive approaches.

**Objectives:**

To systematically evaluate the efficacy and safety of invasive and non-invasive neuromodulation techniques for motor symptom improvement in adults with isolated dystonia.

**Methods:**

A systematic search of PubMed, Embase and Cochrane Library (January 2007–December 2020) was conducted according to PRISMA guidelines. Randomized and non-randomized clinical studies assessing motor outcomes after GPi-DBS, STN-DBS, rTMS or tDCS were included. Risk of bias was assessed using design-specific tools.

**Results:**

Twelve studies met the inclusion criteria. GPi-DBS provided consistent and sustained motor improvement across dystonia phenotypes with an acceptable safety profile. STN-DBS showed comparable efficacy in selected cohorts, with faster early clinical response and lower energy consumption, although the lack of prospective head-to-head trials prevents definitive conclusions. Non-invasive techniques (rTMS and tDCS) yielded modest but clinically meaningful benefits, particularly when combined with structured rehabilitation. Study heterogeneity precluded quantitative synthesis.

**Conclusions:**

Neuromodulation is an effective and safe treatment for isolated dystonia. GPi-DBS remains the best-supported approach, STN-DBS a promising option in selected patients, and non-invasive techniques a potential adjunct. Prospective comparative trials and standardized, phenotype-driven strategies are still needed.

## Introduction

Dystonia is a hyperkinetic movement disorder characterized by sustained or intermittent abnormal movements or postures; movements and postures are patterned and sometimes tremulous or jerky, usually exacerbated by voluntary movements. Dystonia, conceptualized as a complex group of disorders, leads to significant functional impairment, resulting in a remarkable reduction of quality of life [1]. Regardless of body distribution, phenomenology and underlying etiology, clinical management can be complex, especially in patients refractory to botulinum toxin, whose effect is transient and usually exhibits large inter-individual variability, likely underpinned by multiple interacting factors [2, 3].

Over the last decades, neuromodulation has revolutionized the therapeutic approach to dystonia, offering both invasive and non-invasive options; the most established neuromodulatory approach is Deep Brain Stimulation (DBS). DBS is a neurosurgical treatment consisting of implantation of electrodes in specific structures of the brain [4]. DBS is currently FDA and EMA approved for a growing list of neurological disorders, including dystonia [5].

DBS has consistently demonstrated long-term efficacy in dystonia across different phenotypes. Most evidence derives from bilateral stimulation of the globus pallidus internus (GPi), which has shown sustained benefit in patients with isolated generalized and segmental dystonia [6, 7]. Subsequent observational cohorts confirmed these findings: for example, Isaias et al. (2009) reported a motor improvement at one year, maintained over extended follow-up, while Krause et al. [8] documented stable clinical benefit lasting up to 16 years.

Beyond GPi, alternative targets such as the subthalamic nucleus (STN) and the ventral intermediate nucleus of the thalamus (Vim) have also been explored, particularly in focal or cervical dystonia, with encouraging results in selected cases [9, 10]. Both unilateral and bilateral approaches have been attempted, depending on symptom distribution and severity, highlighting the adaptability of DBS to different clinical presentations.

In parallel, non-invasive neuromodulation techniques (NIBS) such as repetitive transcranial magnetic stimulation (rTMS) and transcranial direct current stimulation (tDCS) are emerging.

TMS uses a strong and rapidly changing magnetic field to induce an electric field in the brain, affecting especially neurons located in the crown-lip region of cortical gyri, activating axons in their bends and ramifications [11]. tDCS employs electrodes to deliver electrical currents to the brain; it is mainly neuromodulatory, affecting the resting membrane potential toward depolarization with anodal stimulation and toward hyperpolarization with cathodal stimulation [12]. Controlled clinical studies have shown that these methods, while having more modest effects than DBS, offer significant motor improvements, especially in task-specific focal dystonia, with a high safety profile [13, 14], although overall effect sizes are generally small [15].

Another emerging approach is Magnetic Resonance–Guided Focused Ultrasound (MRgFUS), a non-invasive ablative technique that enables the creation of precise lesions in real time. According to a scoping review by Guinal et al. [16], this technique has demonstrated significant clinical improvements in focal dystonia — such as cervical dystonia and task-specific focal hand dystonia — with an average 29.4% reduction in motor scores on the Burke–Fahn–Marsden Dystonia Rating Scale (BFMDRS) and the Toronto Western Spasmodic Torticollis Rating Scale (TWSTRS).

Despite these advances, the literature remains heterogeneous in terms of experimental design, technical parameters, and outcome measures, necessitating a systematic and comparative evaluation of the efficacy and safety of these techniques. This systematic review seeks to bridge that gap by rigorously appraising studies of DBS, rTMS and tDCS on dystonia, with particular attention to long-term outcomes and their implications for future personalised, multimodal treatment strategies [12, 15].

## Materials and methods

This systematic review was conducted in accordance with the PRISMA guidelines. A comprehensive literature search was performed in PubMed, Scopus, and Web of Science, covering articles published between January 2007 and December 2020. Emerging studies published after 2020 were also considered for contextual discussion but were not included in the quantitative synthesis. Keywords included “dystonia,” “deep brain stimulation,” “transcranial magnetic stimulation,” “transcranial direct current stimulation,” “motor improvement,” and “neuromodulation.”

**Fig. 1:**
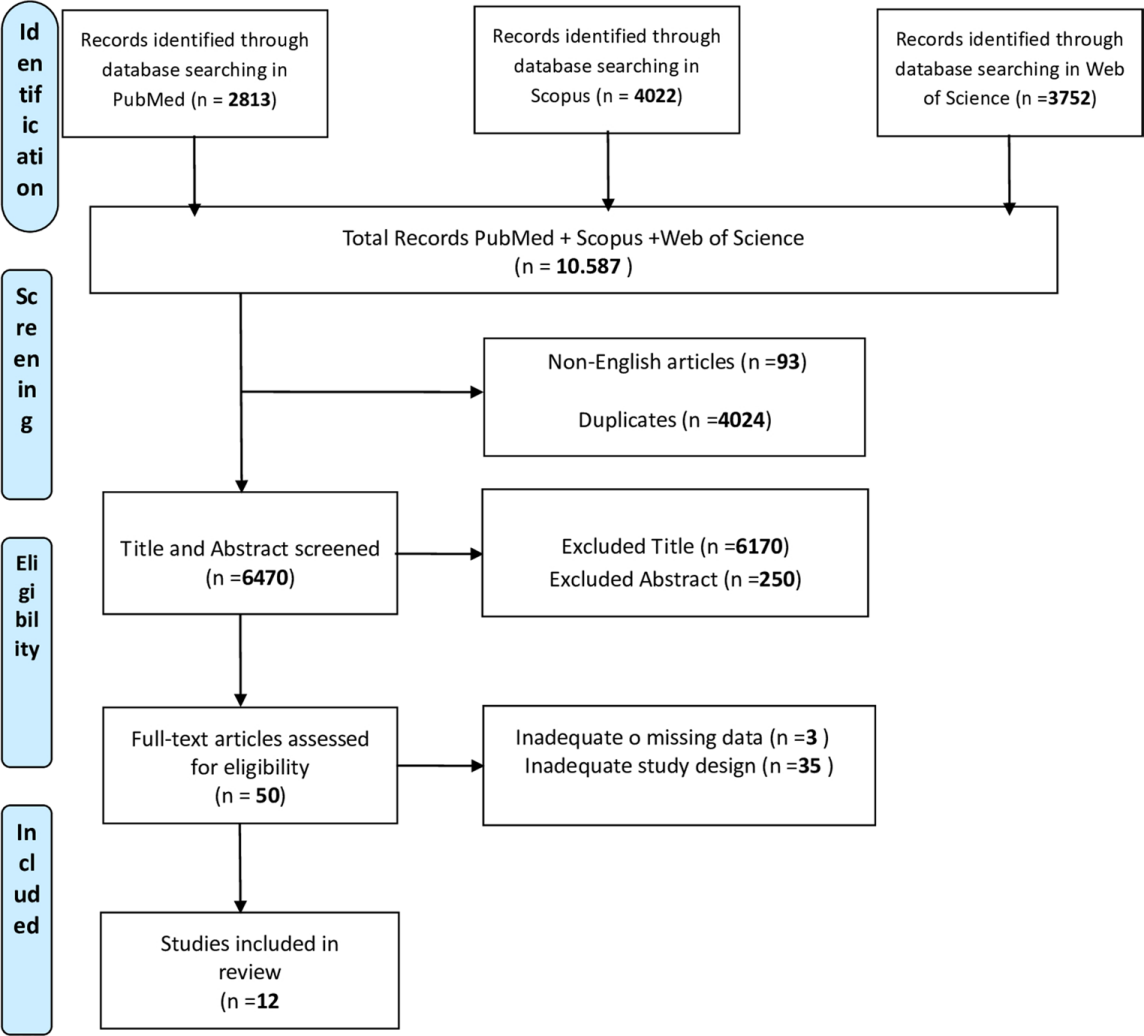
PRISMA flow diagram of study selection

The database search yielded a total of 10,587 citations—2,813 from PubMed, 4,022 from Scopus, and 3,752 from Web of Science. After removing 4,117 duplicates, 6,469 titles and abstracts were screened for relevance (see Fig. 1, PRISMA flowchart). Fifty articles were selected for full-text review, but only twelve studies met all predefined inclusion criteria. All references were cross-checked manually against their original publications to ensure accuracy in authorship, journal source, and bibliographic details. Eligible studies included randomized clinical trials, prospective cohort studies, and case series including at least four adult patients with isolated dystonia (generalized, segmental, focal, or task-specific) refractory to pharmacological treatment. Acceptable interventions were DBS targeting the GPi or STN, as well as non-invasive neuromodulation techniques such as rTMS and tDCS. Only studies with a minimum 6-month follow-up were included. Non-invasive neuromodulation techniques (rTMS and tDCS) were discussed qualitatively, as their follow-up duration was typically shorter than six months.

Exclusion criteria encompassed pediatric populations, secondary dystonia, and studies lacking validated motor outcomes such as the BFMDRS or the TWSTRS or other validated scales.

## Risk of bias assessment

**Fig. 2:**
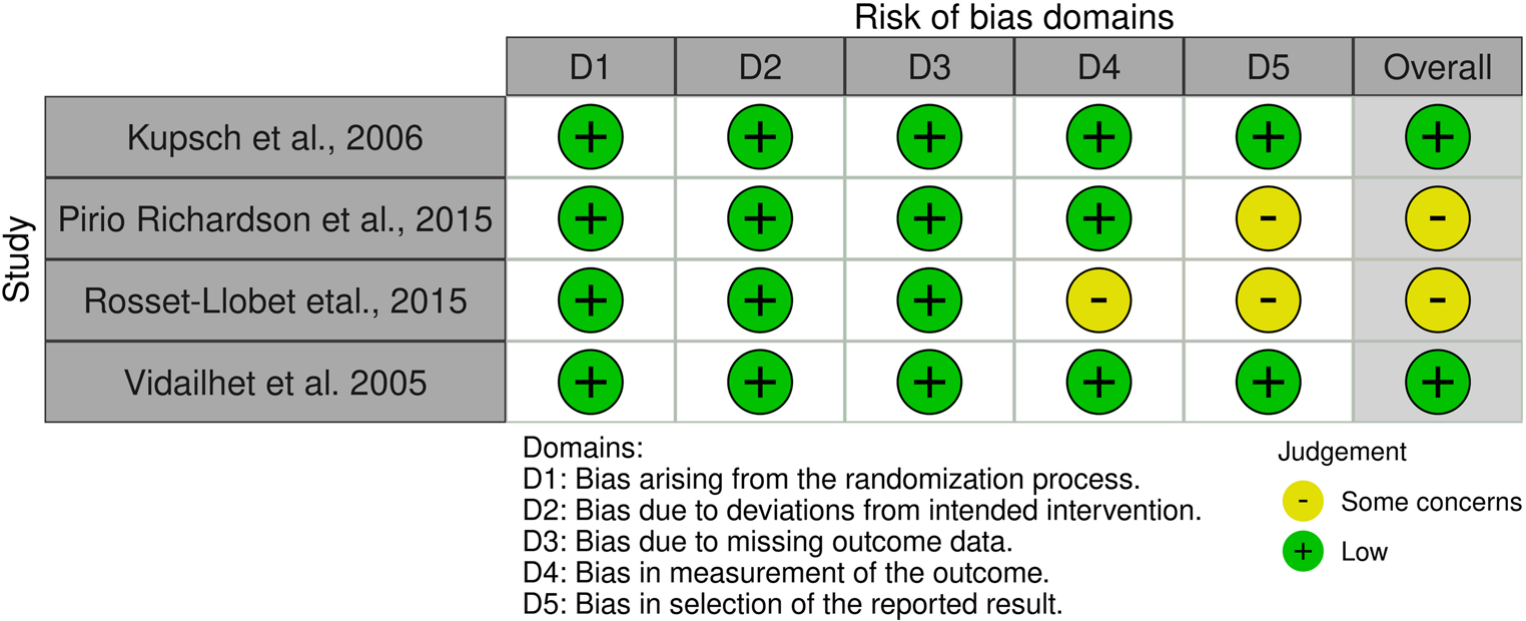
Risk of bias assessment of randomized controlled trials using the RoB 2 tool

**Fig. 3:**
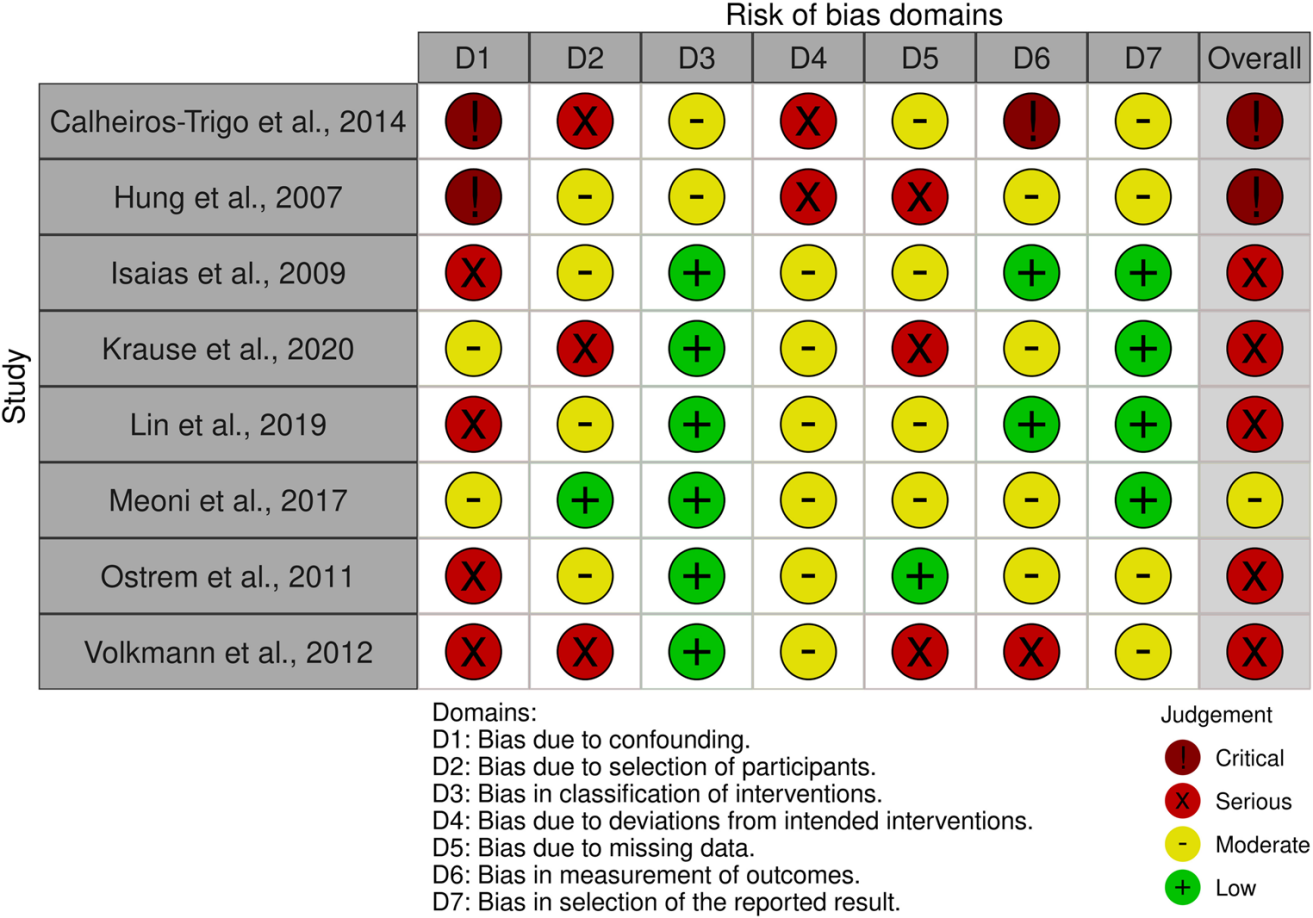
Risk of bias of non-randomized studies assessed using the ROBINS-I tool

The methodological quality of the included studies was assessed using the RoB 2 tool for randomized controlled trials and the ROBINS-I tool for non-randomized studies (Figs. 2 and 3).

### Randomized controlled trials

As shown in Fig. 2, the overall risk of bias among the randomized trials ranged from low to some concerns. Kupsch et al. [6] and Vidailhet et al. [7] were judged to have a low overall risk of bias across all domains, reflecting adequate randomization, blinded video-based outcome assessment, complete outcome data, and prespecified analyses.

Pirio Richardson et al. [13] showed some concerns in the domain related to the selection of the reported results, while Rosset-Llobet et al. [14] presented additional concerns in outcome measurement. Despite these limitations, both studies maintained appropriate blinding and complete outcome data.

### Non-randomized studies

The ROBINS-I assessment (Fig. 3) showed that all non-randomized studies had at least a moderate overall risk of bias.

Meoni et al. [17] was the only study rated as having a moderate overall risk of bias.

A serious overall risk of bias was identified in Isaias et al. [18], Krause et al. [8], Lin et al. [9], Ostrem et al. [10], and Volkmann et al. [19], mainly due to confounding, non-random participant selection, and incomplete follow-up. In Volkmann et al., additional concerns were related to missing outcome data and open-label outcome assessment.

Calheiros-Trigo et al. [20] and Hung et al. [21] were judged to have a critical overall risk of bias, driven primarily by critical confounding in small uncontrolled cohorts.

### Implications for evidence synthesis

Overall, the risk of bias demonstrated a clear gradient according to study design. Low-risk evidence was limited to two randomized trials, whereas most long-term data were derived from studies with serious or critical risk of bias. These methodological limitations precluded quantitative pooling and required cautious interpretation of comparative effectiveness.

## Results

This systematic review included twelve original studies that met all inclusion criteria, comprised more than two hundred adult patients diagnosed with isolated or task-specific dystonia (Table 1).

**Table 1:** Summary of findings from included studies

References	Aim	Study Design	Simple Size	Intervention	Assessment	Main Results
[20]	Evaluate the efficacy of GPi‑DBS in medically refractory cervical dystonia.	Retrospective case series; 12‑month follow‑up.	4 patients with idiopathic cervical dystonia.	Bilateral GPi deep brain stimulation.	TWSTRS (severity & disability) at baseline, 3, 6, 12 months.	Mean TWSTRS improvement: ~74% severity, ~80% disability at 12 months; no stimulation‑related adverse effects.
[21]	Evaluate long‑term efficacy of bilateral GPi‑DBS in primary cervical dystonia.	Retrospective case series; follow‑up 12–67 months.	10 patients with medically refractory cervical dystonia.	Bilateral GPi deep brain stimulation; standardized programming.	TWSTRS (severity, disability, pain); clinical follow‑up; AE monitoring.	Severity ↓ 54.8%, disability ↓ 59.1%, pain ↓ 50.4% at last follow‑up; stable benefit; few stimulation‑related AEs; one infection requiring electrode replacement.
[18]	Assess long‑term safety and efficacy of GPi‑DBS in primary generalized dystonia.	Retrospective case series with annual follow‑up up to 8 years.	30 patients (28 bilateral DBS, 2 unilateral).	GPi deep brain stimulation; two stimulation protocols (130 Hz vs 60 Hz).	BFMDRS‑Motor & Disability; blinded video scoring; adverse events; TEED; IPG longevity.	~80% sustained motor improvement; disability ↑ ~70–75%; low hardware AE rate; 60 Hz preserved battery life with equal efficacy.
[8]	Evaluate long‑term motor, disability, mood, and QoL outcomes after bilateral GPi‑DBS in isolated dystonia.	Retrospective observational study; long‑term follow‑up 8–16 years.	36 eligible; 19 completed long‑term follow‑up.	Bilateral GPi deep brain stimulation; standard programming; long‑term adjustments as needed.	BFMDRS (motor & disability), TWSTRS/Tsui (for CD), SF‑36, BDI, adverse events, electrode localization.	Motor improvement ~68% long‑term; disability ~54%; QoL ↑ (physical domains); mood ↑ (BDI ↓ 37%); stable benefit up to 16 years; stimulation‑related AEs mild/modifiable; hardware infections in some cases.
[6]	Test whether bilateral GPi‑DBS is superior to sham stimulation in primary generalized/segmental dystonia.	Randomized, double‑blind, sham‑controlled trial + open‑label extension.	40 implanted; 20 active vs 20 sham in blinded phase.	Bilateral GPi deep brain stimulation; standardized programming; sham = 0 V.	BFMDRS‑Movement & Disability (blinded video ratings), SF‑36, VAS pain/dystonia, psychiatric scales, AE monitoring.	At 3 months: movement ↓ 39% with DBS vs 5% with sham; disability ↓ 38% vs 8%; QoL ↑; benefits sustained at 6 months; AEs mostly device‑related but manageable.
[9]	Compare GPi vs STN DBS effectiveness in isolated dystonia.	Matched retrospective cohort; 12‑month follow‑up.	30 patients (14 GPi, 16 STN).	Bilateral GPi or STN DBS; standardized programming.	BFMDRS (motor & disability), SF‑36, TEED, blinded video scoring.	Both targets effective; STN shows faster early improvement; GPi better for axial symptoms; STN uses less energy (lower TEED).
[17]	Assess long‑term efficacy and safety of GPi‑DBS across idiopathic, inherited, and acquired dystonia.	Retrospective cohort; long‑term follow‑up up to 20 years.	61 patients (IID, INH, AD).	Bilateral GPi deep brain stimulation; standardized programming.	BFMDRS‑Motor & Disability at baseline, 1‑year, and last follow‑up; AE monitoring; medication changes.	IID/INH: motor improvement ~58% sustained long‑term; disability improvement ~23–37%. AD: modest but stable motor benefit (~14–17%). Hardware AEs common; stimulation parameters stable over time.
[10]	Evaluate safety, efficacy, QoL, and neuropsychological effects of bilateral STN‑DBS in primary cervical dystonia.	Prospective pilot study with blinded ratings; 12‑month follow‑up.	9 patients with medically refractory primary cervical dystonia.	Bilateral STN deep brain stimulation; standardized programming.	TWSTRS (blinded), BFMDRS, JBOS, SF‑36, CGI, neuropsychological battery, AE monitoring.	TWSTRS improved ~63% at 12 months; QoL ↑; no bradykinesia; transient dyskinesia common but manageable; no cognitive decline; some depression/weight gain.
[13]	Identify optimal rTMS cortical targets and assess safety/tolerability in cervical dystonia.	Randomized, sham‑controlled, blinded pilot trial; crossover across 5 stimulation sites.	8 completed (9 enrolled).	Low‑frequency (0.2 Hz) rTMS over MC, dPM, SMA, ACC, and sham; 15‑min sessions.	TWSTRS severity (blinded), neurophysiology (dPMI, CSP), tolerability, AE monitoring.	Greatest improvement after dPM and MC stimulation; cumulative TWSTRS improvement across sessions; treatment safe and well‑tolerated; no significant neurophysiology changes.
[14]	Test whether biparietal tDCS enhances the effectiveness of sensory‑motor retuning (SMR) in task‑specific focal hand dystonia.	Parallel, double‑blind, randomized controlled trial (real vs sham tDCS).	30 musicians with right‑hand TSFHD.	2‑week SMR program + either real or sham biparietal tDCS (20 min, 2 mA).	Dystonia Severity Rating (15‑item task‑based test); therapist’s blinded evaluation; AE monitoring.	Both groups improved, but real tDCS produced significantly greater gains; no adverse effects; tDCS enhanced SMR effectiveness.
[7]	Evaluate efficacy and safety of bilateral GPi‑DBS in primary generalized dystonia.	Prospective multicenter controlled study with blinded assessments.	22 adults with primary generalized dystonia.	Bilateral GPi deep brain stimulation; standardized programming.	BFMDRS (movement & disability), SF‑36, MMSE, BDI; blinded video ratings.	Movement ↓ 51%, disability ↓ 44% at 12 months; QoL ↑; cognition/mood unchanged; few reversible AEs.
[19]	Evaluate long‑term (5‑year) efficacy and safety of GPi‑DBS in primary generalized or segmental dystonia.	Prospective multicenter randomized trial + 5‑year open‑label extension.	40 implanted; 38 entered long‑term follow‑up; 32 completed 5‑year visit.	Bilateral GPi deep brain stimulation; initial sham vs active phase, then all active stimulation.	BFMDRS‑Motor & Disability; pain; QoL (SF‑36); psychiatric scales; AE monitoring.	Motor improvement ~58–67% sustained at 3–5 years; disability and QoL improved; serious AEs mostly device‑related but resolved; overall long‑term efficacy maintained.

The selected studies assessed the clinical efficacy of neuromodulation techniques, both invasive—such as DBS and non-invasive approaches, including rTMS and tDCS. Most of the available evidence focused on DBS, with the GPi emerging as the most consistently effective target for generalized and segmental forms of isolated dystonia. Early support for the long-term benefit of GPi-DBS came from Isaias et al. [18], together with Kupsch et al. [6] and Vidailhet et al. [7], who reported substantial motor improvements one year after surgery that were maintained over time. These findings were later reinforced by Krause et al. [8], whose follow-up ranged from eight to sixteen years and confirmed both stable motor outcomes and sustained improvements in quality of life in patients with generalized, segmental, and cervical dystonia. The multicentre randomized controlled trial conducted by Volkmann et al. [19] further supported these observations, showing a significant motor benefit after just three months of active stimulation, which continued to improve over a five-year period. Meoni et al. [17] further refined the debate, showing that treatment response depends on etiology: patients with idiopathic or genetically defined isolated dystonia experienced markedly greater improvement than those with acquired or combined forms (e.g., dystonia associated with other neurological symptoms such as spinocerebellar ataxia).

Other studies investigated the use of STN as an alternative DBS target, especially in patients with cervical or isolated dystonia. Lin et al. [9] performed a direct comparison between STN and GPi stimulation, reporting a faster clinical response and better energy efficiency with STN, although GPi remained superior for managing axial symptoms. These observations were in line with findings by Ostrem et al. [10], who also evaluated STN-DBS and reported good tolerability, with only transient psychiatric side effects in a minority of patients. Overall, DBS has been attempted in both unilateral and bilateral approaches, and across different targets (GPi, STN, Vim), in various dystonia phenotypes.

Both Calheiros-Trigo et al. [20] and Hung et al. [21] reported significant improvements after GPi-DBS in patients with focal forms of dystonia, especially cervical dystonia. Although based on small samples, these studies demonstrated clinically meaningful reductions in symptom severity, with sustained benefits and minimal adverse effects over follow-up periods extending beyond two years.

In the domain of non-invasive neuromodulation, two studies were included. Pirio Richardson et al. [13] assessed low frequency rTMS of motor and premotor cortices in patients with cervical dystonia, revealing a statistically significant reduction in TWSTRS scores. No major adverse events were reported, indicating good tolerability of the treatment. At the same time, Rosset-Llobet et al. [14] investigated the use of bi-parietal tDCS combined with sensorimotor retraining in musicians affected by task-specific focal hand dystonia. Their findings indicated superior functional improvement in the active stimulation group compared to sham, highlighting the potential of integrated rehabilitation and neuromodulation protocols in treating maladaptive cortical plasticity. Nevertheless, both rTMS and tDCS studies were limited by small sample sizes, high variability, and low effect sizes [13, 14], which explains why non-invasive neuromodulation is not yet considered a standardized treatment option.

## Discussion

The results of this systematic review support the evidence that neuromodulation techniques are effective in treating various forms of isolated and task-specific dystonia. Among the included studies, deep brain stimulation of GPi emerged as the most consistently effective intervention, with multiple studies reporting significant and long-lasting improvements in motor function and quality of life.

This review also highlighted the therapeutic potential of STN as an alternative target in DBS, particularly in patients with cervical dystonia. Studies such as Lin et al. [9] and Ostrem et al. [10] showed that STN-DBS may induce faster clinical responses and reduce energy consumption, a relevant consideration for device sustainability. However, these advantages must be carefully evaluated with possible risk of transient neuropsychiatric effects. Consequently, target selection should be individualized based on symptom distribution, patient phenotype, and long-term therapeutic outcomes. Although retrospective comparisons [9] suggest a faster early response and lower energy consumption with STN-DBS, the absence of prospective, randomized target-to-target trials prevents any definitive conclusion. These findings should therefore be interpreted as preliminary and hypothesis-generating rather than comparative evidence.

The evidence from studies on non-invasive brain stimulation, including repetitive transcranial magnetic stimulation (rTMS) and transcranial direct current stimulation (tDCS), should not be framed as “alternatives” to DBS given the limited evidence base (one study per technique meeting inclusion criteria), particularly in focal dystonia. Although the magnitude of improvement reported in these studies is modest (e.g., small reductions in TWSTRS severity after low-frequency rTMS; functional gains in task-specific hand dystonia with bi-parietal tDCS combined with sensorimotor retraining), their safety profile and feasibility make them suitable for patients who are not eligible for surgery or prefer less invasive approaches. Notably, Rosset-Llobet et al. [14] demonstrated that combining tDCS with sensorimotor retraining resulted in significant clinical improvement in musicians with task-specific hand dystonia, highlighting the potential of integrative neuromodulation and rehabilitative strategies. Nonetheless, high inter-study variability, small sample sizes, and low effect sizes help explain why non-invasive neuromodulation has not yet been incorporated into standardized treatment pathways. Overall, these observations are consistent with the broader meta-analytic evidence reported by Morrison-Ham et al. [22], who found that non-invasive brain stimulation produces only modest overall effects, largely shaped by stimulation parameters and by the integration of concurrent motor training.

Overall, while GPi-DBS remains the gold standard for severe, medication-refractory dystonia, this review emphasizes the need for a patient-centred approach to neuromodulation. Decisions regarding target selection, procedural invasiveness, and integration with rehabilitation should be adopted to each patient’s clinical profile. Increasingly, predictive models and individualized algorithms—incorporating neuroimaging and genetic data— could be useful to refine treatment planning.

Recent advances in network-based targeting have increased our understanding of the most effective stimulation strategies. Lesion network mapping has shown that diverse lesion sites causing cervical dystonia to converge on a shared functional network involving cerebellum and somatosensory cortex, with effective DBS sites connected to these same regions [23]. Similarly, probabilistic mapping of pallidal stimulation identified a “sweet spot” in the ventroposterior GPi and adjacent sub pallidal white matter, enabling predictive modelling of individual outcomes [24]. Connectivity-based approaches also underscore the importance of anticipating side effects. For example, stimulation sites linked to cognitive decline in Parkinson’s disease were more connected to limbic and cerebellar cognitive regions—suggesting that similar principles could help minimize risks in dystonia [24]. Circuit-informed modelling has revealed phenotype-specific pathways: optimal stimulation for cervical dystonia modulates the striatopallidofugal tract, while generalized dystonia responds best to pallidothalamic bundle modulation. Both phenotypes share a common network involving the cerebellum and somatomotor cortex [25]. Finally, structural MRI–based cortical fingerprints have demonstrated robust predictive value: patients with preserved integrity in sensorimotor and visuomotor regions tend to respond more favourably to DBS, while those with cortical atrophy show poorer outcomes. Machine learning classifiers trained on these imaging features have reached up to 88% accuracy in forecasting treatment response, reinforcing their potential role in clinical stratification [15]. Collectively, these findings show how connectomic and imaging-based approaches can refine patient selection, guide target optimization, and help anticipate both therapeutic benefits and potential risks—paving the way for a true paradigm shift toward precision neuromodulation. Despite these encouraging findings, their interpretation should remain cautious in light of the methodological limitations that persist across studies. Even among randomized trials, such as that by Rosset-Llobet et al. [14], small sample sizes, exploratory designs, and the absence of preregistered statistical plans are still common shortcomings. These gaps underscore the need for future studies to adopt more rigorous and standardized methodologies, including that novel neuromodulation strategies.

In summary, current data point toward a gradual transition to a multimodal and personalized model of dystonia care. The integration of well-established surgical interventions such as DBS with non-invasive neuromodulation techniques and individualized neurorehabilitation could maximize therapeutic outcomes and durable functional recovery. Continuous refinement of clinical protocols, together with the adoption of predictive and imaging-based tools, will be key to advancing true precision medicine in this field.

### Emerging techniques outside the review timeframe (MRgFUS)

Although MR-guided focused ultrasound (MRgFUS) was not included in the formal systematic review due to the predefined timeframe, its recent emergence in the dystonia field warrants discussion. MRgFUS is a non-incisional but irreversible ablative technique that allows precise lesioning of deep brain structures under real-time MRI guidance. In a recent scoping review, Guinal et al. [16] summarized the early clinical experience across eleven studies and reported meaningful improvements in several focal dystonia subtypes—including cervical dystonia, writer’s cramp, and X-linked dystonia-parkinsonism. On average, motor symptoms improved by approximately 29% on validated scales such as the BFMDRS and TWSTRS, and patients frequently reported parallel gains in daily functioning and symptom burden.

The safety profile described in these early reports appears acceptable, with adverse effects such as dysarthria, gait imbalance, or transient paraesthesia generally resolving over time. Nonetheless, the irreversible nature of the lesion requires careful patient selection, particularly in comparison with DBS, which offers the advantage of adjustability and reversibility. MRgFUS also presents technical constraints: skull density variability can limit ultrasound transmission, acoustic windows restrict target accessibility, and long-term follow-up data remain scarce. For these reasons, despite its appeal—immediate clinical effect, absence of implanted hardware, and elimination of device-related complications—MRgFUS should currently be regarded as a promising but still experimental option. Larger, controlled studies are needed to clarify optimal targets, durability of benefit, and its potential role alongside or in place of established neuromodulation strategies [16].

### Clinical implications and future perspectives

From a clinical standpoint, the evidence synthesized in this review reinforces GPi-DBS as the most reliable and well-established therapeutic option for adults with severe, medication-refractory isolated dystonia. Its long-term efficacy—documented across generalized, segmental, and focal phenotypes—makes GPi the preferred target in routine practice, particularly when durable symptom control and broad motor improvement are the primary goals.

STN-DBS, while supported by a smaller and more heterogeneous body of evidence, represents a reasonable alternative in carefully selected patients, especially those with focal or cervical dystonia who may benefit from a faster clinical response or lower energy consumption. However, in the absence of prospective, controlled comparisons between GPi and STN, these potential advantages should be interpreted with caution. Target selection must therefore remain individualized, guided by symptom distribution, patient phenotype, comorbidities, and long-term therapeutic expectations.

Non-invasive neuromodulation techniques, including rTMS and tDCS, may serve as adjunctive or exploratory options for patients who are not suitable candidates for surgery or who may benefit from interventions aimed at modulating maladaptive plasticity. Although their clinical effects are modest, their safety, feasibility, and compatibility with structured rehabilitation make them valuable components of a broader multimodal treatment strategy. In particular, the combination of NIBS with sensorimotor retraining appears promising for task-specific dystonia, where maladaptive learning mechanisms play a central role.

Emerging technologies such as MR-guided focused ultrasound (MRgFUS) may further expand the therapeutic landscape, particularly for patients who are not ideal candidates for conventional DBS or who prefer non-implantable approaches. While still experimental, MRgFUS offers immediate post-procedural effects and avoids hardware-related complications, though its irreversible nature and technical constraints require careful patient selection.

Looking ahead, the field is moving toward increasingly personalized neuromodulation strategies. Advances in connectomics, neuroimaging biomarkers, and machine-learning–based predictive tools hold promise for refining patient selection, optimizing target choice, and anticipating treatment response. Although adaptive and closed-loop DBS systems were not part of the studies included in this review, they represent compelling avenues for future research, offering the possibility of real-time, biomarker-guided modulation of dystonic circuits. Integrating these innovations with established surgical and rehabilitative approaches will be essential to achieving a truly multimodal, patient-centred model of dystonia care.

Overall, current evidence encourages neurologists to move beyond a “one-target-fits-all” paradigm and toward a more nuanced, phenotype-driven approach—one that combines established interventions like GPi-DBS with emerging technologies, individualized rehabilitation, and network-informed decision-making to optimize long-term outcomes.

## Conclusion

Neuromodulation has progressively established itself as a valuable and generally safe therapeutic avenue for isolated and task-specific dystonia. Among invasive interventions, deep brain stimulation of the globus pallidus internus (GPi) continues to offer the most consistent and durable benefits, particularly in generalized and segmental forms. Stimulation of the subthalamic nucleus (STN) has also produced encouraging results—especially in cervical dystonia—characterized by a faster onset of clinical improvement and reduced energy consumption, although long-term comparative evidence remains limited.

Non-invasive approaches such as rTMS and tDCS provide additional opportunities for symptom modulation, especially in focal and task-specific dystonia. Their excellent safety profile and procedural simplicity make them appealing for individuals who are not candidates for surgery or who prefer non-surgical options. Nonetheless, the modest effect sizes and methodological variability across studies currently limit their widespread clinical adoption.

MR-guided focused ultrasound (MRgFUS) is emerging as a promising incisionless alternative, with early studies reporting meaningful improvements in focal dystonia and the advantage of immediate post-procedural effects. The absence of implanted hardware further distinguishes this technique, although its irreversible nature and the scarcity of long-term data underscore the need for larger, controlled investigations.

As the field evolves, dystonia management is shifting toward a more personalized and multimodal framework. Integrating clinical phenotype, neuroanatomical targeting, and structured rehabilitation is becoming increasingly central to optimizing outcomes. Advances in connectomic modeling, neuroimaging biomarkers, and machine-learning–based predictive tools are poised to refine patient selection and guide therapeutic decision-making with greater precision. With these developments, neuromodulation is expected to transition from a uniform treatment paradigm to a tailored, circuit-informed approach that aligns interventions with each patient’s unique neural architecture and functional needs.

## Legend

ACC: Anterior Cingulate Cortex
AD: Acquired Dystonia
AE / AEs: Adverse Event(s)
BDI: Beck Depression Inventory
BFMDRS: Burke–Fahn–Marsden Dystonia Rating Scale
CD: Cervical Dystonia
CGI: Clinical Global Impression
CSP: Cortical Silent Period
DBS: Deep Brain Stimulation
dPM: Dorsal Premotor Cortex
dPMI: Dorsal Premotor Inhibition
GPi: Globus Pallidus Internus
IID: Idiopathic Isolated Dystonia
INH: Inherited Dystonia
IPG: Implantable Pulse Generator
JBOS: Jankovic Botulinum toxin Outcome Scale
MC: Motor Cortex
MMSE: Mini-Mental State Examination
QoL: Quality of Life
RCT: Randomized Controlled Trial
rTMS: Repetitive Transcranial Magnetic Stimulation
SF-36: Short Form 36 Health Survey
SMA: Supplementary Motor Area
SMR: Sensory-Motor Retuning
STN: Subthalamic Nucleus
tDCS: Transcranial Direct Current Stimulation
TEED: Total Electrical Energy Delivered
TSFHD: Task-Specific Focal Hand Dystonia
Tsui score: Tsui Scale for Cervical Dystonia
TWSTRS: Toronto Western Spasmodic Torticollis Rating Scale
VAS: Visual Analog Scale
